# Buffalo Medical and Surgical Association

**Published:** 1883-09

**Authors:** W. H. Heath

**Affiliations:** Secretary pro tem.


### Society Reports.

### Buffalo Medical and Surgical Association.

*Regular Meeting, August 7th, 1883.*

Present—Drs. Hopkins, Davidson, Long, Eli Long, Hubbell, Ingraham, Heath, Cronyn, Hauenstein, Coakley, Howe, Hayd, Montgomery, Thornton, Brecht, Crego.

In the absence of C. M. Daniels, Secretary, Dr. W. H. Heath acted as Secretary.

After some discussion relative to the mode of election of new members, Dr. Eli H. Long and Dr. Packwood were elected members.

Paper entitled "Medical Education" was read by Dr. Floyd Crego.

*Discussion*—Dr. Davidson was glad to express his interest and thanks, spoke of the importance of the subject; took exception to the expression of Dr. Crego that "one college" was the exception to defective system existing in all others, and mentioned the fact that the University of Pennsylvania, in face of strong opposition, took a new departure and strong stand for higher education, and was to-day reaping the honor and benefit of it. Of the necessity for a State Medical Board, there should be no difference of opinion; thought it was absolutely idle to look to medical colleges for any improvement. When the American medical colleges met and formed an association, something better was looked for in the way of an advance toward a higher standard; but, as is well known, among the first colleges to withdraw was one of the most prominent in this State, and from which better action had been expected. Therefore, the profession itself should act and take up the matter, and urge and have a State Board of Medical Examiners appointed, before whom all should appear and pass an examination before being allowed to practice. Its formation and appointment were important considerations. But one point was vital to it, that it should be free from all and every connection with the medical schools; it would be a mistake for any professor of a college to be a member of such board. Dr. Davidson thought it a great credit to the Erie County Medical Society to be one of the first to take the initiative. Owing to the fact that the bill making such law would be strongly opposed, the whole profession should be aroused, and the united action of all legal medical bodies invoked.

Dr. Hopkins had no apology to offer for being interested or speaking on so important a matter. Any one who has the honor and interest of his calling at heart, who feels and considers it a noble profession and not a trade, must certainly feel humiliated at the relations existing between the profession and the people, for it certainly has no hold whatever on the confidence of the public, who consider it largely in the light of a strong sect; was glad Dr. Crego laid the blame upon the profession, where it belonged. Dr. Hopkins referred to the distaste Americans have to submit to regulations, and their high sense of individual liberty. He thought, however, the people were gradually awakening to the importance of educated physicians and an improvement on present systems; referred to the establishment of quarantines, and thought and hoped the time would soon come when they will demand a profession who will scrutinize them as effectually as quarantine now scrutinizes contagious diseases, etc. He thought people should be educated, and thought that educated medical men were a *necessity* to the State.

Dr. Hubbell thought the proposed Medical Board of Examiners should be free from all cliques, and particularly college cliques. It should be broad-minded and avoid sectarianism; did not think any bill to establish the proposed board would be passed by legislature if at all sectarian in character, and he feared the present bill, as standing, rather inclined that way.

Dr. Hauenstein said it was an admitted fact that higher medical education was a necessity; thought there were so many opinions as to how the result was to be reached, and that there was a great difficulty. If some form could be given the matter it would then be easier to accomplish the object. So long as different members of the profession urge particular views but little can be accomplished. Harmony and unity should be given the matter. Thought the responsibility of the present state and its improvement rested with the profession itself, and not with the laity. It was their duty, and if definite form could be given to it and it was properly agitated, it could soon be accomplished.

Dr. Coakly spoke of the difficulty of obtaining the attention of the legislature, or even a committee. He had had experience in the matter in another State, and found it very discouraging. Found intelligent city representatives willing, but that the rural ones were indifferent, ignorant, and didn't believe in it. He hoped much for the bill in this State.

Dr. Howe spoke of the importance of the subject and regretted that time rendered discussion so superficial and limited, leaving many points untouched. The paper, condensed, referred to three faults:

1st. Physicians who take in illiterate and ignorant students. He would like to hear the experience of the censors in examining many of these men, and what they have to say about it.

2d. The colleges and their abuses, etc.

3d. State Board of Examiners and the want of it, which is so important. Yet, in looking back one hundred years, there was, he thought, a great advance; but a little over that time surgeons and barbers were one.

Dr. H. referred to the peculiar factors at work in this county, notably the absence of strong government to guide us. Referred to the necessity of teaching physiology in public schools, and educating the rising generation.

Dr. Hayd thought the public felt they were not properly protected. He considered the medical colleges of the United States too mercenary, and that they were established just like factories and glucose works, purely for profit. Referred to short term of two years. Compared the American system with the European and Canadian, to the disparagement of the former. Spoke of State Board of Examiners in Canada, and how they were conducted, and their good results in regulating practice, etc.

Dr. Heath referred to the University of Pennsylvania, which had, some years ago, established a higher standard, graded course, etc. Spoke of over-crowded state of profession in the United States, and stated that there was no other business or profession where so few earned even a living, where competition was so cheap and successful.

As an instance of the poor medical education furnished, said examining boards of United States army, navy and marine hospital services, rejected about eighty per cent. of all applicants.

Called attention to the fact that, without exception, the United States was the only country where medical education was conducted without the principles of preliminary examination, graded and extended course, and examination by disinterested persons. Mentioned the well-known want of confidence in the profession, etc.

Dr. Cronyn wound up the discussion by saying the least the colleges could do was to require a preliminary examination, and extend the course to nine months, at least. Compared the teaching of other professions, law and divinity, where preliminary education is required. Also referred to the resources a skilled medical man should have. Lawyers and clerics could think out and prepare their cases, and take their time to it. With medical men, they had often to have their resources at the fingers' ends, and take action at once to save life and health—no time for reference to authorities, libraries and counsel. Thought, however, on the whole, that medical men are improving. He lamented that the government did not see the necessity of protecting the health of its people, as well as its property, by legislation, and regulating medical colleges and medical practice. If the medical bill referred to would pass, many causes of complaint will be set aside. Referred, also, to importance of careful technical education, preliminary examinations, long courses, and the great difference between our system and that of foreign and Canadian governments.

A discussion followed between Crego, Howe, Heath and others, as to how recently barbers and surgeons were one.

Dr. Howe presented a rabbit with a cicatrizing corneal ulcer, healing under use of eserine, the active principle of calabar bean; said that although it was no new thing, having been used in corneal ulcer for six years and more, as yet the exact indications of its use in this very disease were not clear, and it was difficult to decide when to use "atropine" and dilate the pupil, or eserine and contract. In some cases of extensive corneal ulceration, where atropine would seem to be indicated, to get the iris out of the way for prolapsing by dilating, eserine had proved very good by producing the reverse.

Dr. Howe also referred to the power of eserine to reduce inflammation in corneal ulceration, and thought it had somewhat of a specific effect.

Dr. Hubbell spoke of his operations in the use of the drug. Thought atropia better than eserine, which has disappointed him somewhat.

The indications for its use, he thought, were theoretically, at least, clear; that when the ulcer was towards the periphery of cornea, eserine was indicated to draw the iris away and prevent hernia of it at the point of perforation. If, on the other hand, the ulcer were central and threatened perforation, atropia was indicated to draw the iris away.

As to the specific curative value of eserine in corneal ulcer, he has doubts upon it. In view of large numbers of corneal ulcers, the importance of the subject was apparent.

*Prevailing Diseases*—Dr. Hopkins: Gastro-intestinal trouble, with peculiar constitutional sympathy preceding the attack. Chill and fever, with temperature 102–104°, and, in twenty-four hours, the evacuations—a condition he had not noticed before. Other members spoke of frequency of dysentery and diarrhœal troubles and malaria.

W. H. Heath, M. D., *Secretary pro tem.*

